# Supplementary material for: Fluorescence from a single-molecule probe directly attached to a plasmonic STM tip
Source: Nat Commun. 2024 Nov 10;15:9733. doi: 10.1038/s41467-024-53707-2 (PMC11551166; doi:10.1038/s41467-024-53707-2)
Supplement: Supplementary file 1 — Supplementary Information [file 41467_2024_53707_MOESM1_ESM.pdf]

Supplementary Information for Fluorescence from a single molecule probe directly attached to a plasmonic STM tip

Niklas Friedrich,[1,10,*] Anna Rosławska,[2] Xabier Arrieta,[3] Katharina Kaiser,[2,4] Michelangelo Romeo,[2] Eric Le Moal,[5] Fabrice Scheurer,[2] Javier Aizpurua,[6,7,8] Andrei G. Borisov,[5] Tomáš Neuman,[5,9,†] and Guillaume Schull[2,‡]

[1]*CIC nanoGUNE-BRTA, 20018 Donostia-San Sebastián, Spain*
[2]*Université de Strasbourg, CNRS, IPCMS, UMR 7504, F-67000 Strasbourg, France*
[3]*Materials Physics Center, CSIC-UPV/EHU, 20018 Donostia-San Sebastián, Spain.*
[4]*IV. Physical Institute - Solids and Nanostructures, Georg-August-Universität Göttingen, 37077 Göttingen, Germany*
[5]*Université Paris-Saclay, CNRS, Institut des Sciences Moléculaires d'Orsay, 91405, Orsay, France*
[6]*Donostia International Physics Center, 20018 Donostia-San Sebastián, Spain.*
[7]*Department of Electricity and Electronics, FCT-ZTF, UPV/EHU, Leioa 48940, Spain*
[8]*IKERBASQUE, Basque Foundation for Science, Euskadi Plaza 5, Bilbao 48009, Spain.*
[9]*Institute of Physics, Czech Academy of Sciences, Cukrovarnická 10, 16200 Prague, Czech Republic*
[10]*Present address: Institute of Experimental and Applied Physics, University of Regensburg, 93053 Regensburg, Germany*

CONTENTS

Suppl. Note 1. Additional experimental data 2
1. Plasmon corresponding to Fig. 2a 2
2. STML spectra on 4 ML NaCl 3
3. Impact of tip-surface separation and bias voltage on the zero phonon line energy for a PTCDA-tip on top of 2 ML NaCl 4
4. Spectra acquisition parameters 5
5. Adatom cluster imaged with a PTCDA functionalized tip 6

Suppl. Note 2. Theory 7
1. (Time-dependent) density-functional-theory calculations 7
2. Exciton decay rate 8
3. Charge transfer dynamics 8
   1. The wave packet propagation approach 8
4. Analysis of the charge transfer process 10
5. Influence of the protrusion geometry on the plasmon-induced broadening 11
6. Vibronic features in the emission spectrum 11
7. Influence of tilting of the molecule on calculated rates 12
8. Excitation mechanisms of suspended molecules 15
9. Effects of dielectric environment 17

Supplementary references 18

* niklas.friedrich@ur.de
† neuman@fzu.cz
‡ guillaume.schull@ipcms.unistra.fr

Suppl. Note 1. ADDITIONAL EXPERIMENTAL DATA

1. Plasmon corresponding to Fig. 2a

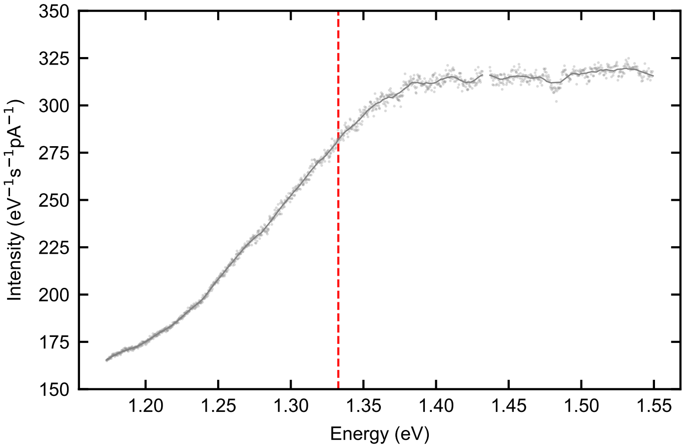

Suppl. Fig. 1. **Plasmon of clean metal tip.** STML spectrum revealing the spectral response of the Ag tip- Ag substrate nanocavity plasmon used to collect the data presented in Fig. 2a. The spectrum was acquired with $V = -2.5\,\mathrm{V}$, $I = 100\,\mathrm{pA}$, $t = 3\,\mathrm{min}$. The red dashed line indicates the emission energy of X^-. Source data are provided as a Source Data file.

2. STML spectra on 4 ML NaCl

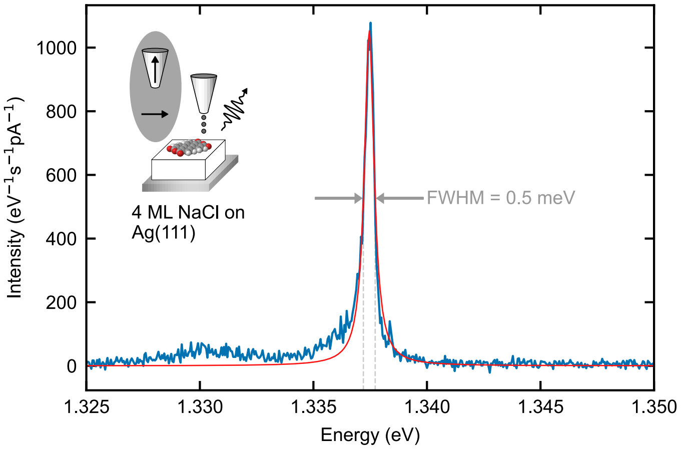

Suppl. Fig. 2. **Minimal fluorescence linewidth.** STML spectrum of a PTCDA molecule adsorbed flat on 4 ML NaCl/Ag(111) (blue curve) obtained with a 1200 lines per mm grating and a reduced spectrometer slit opening to optimize spectral resolution ($\approx 0.22\,$meV). The FWHM $= 0.5\,$meV of the main resonance is extracted from a Lorentzian fit (red curve). The spectrum was acquired with $V = -2.5\,$V, $I = 50\,$pA, $t = 5\,$min. Source data are provided as a Source Data file.

3. Impact of tip-surface separation and bias voltage on the zero phonon line energy for a PTCDA-tip on top of 2 ML NaCl

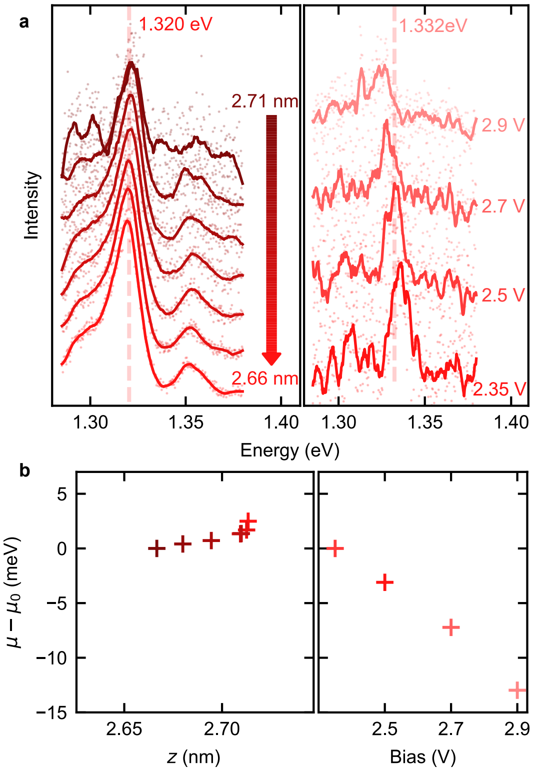

Suppl. Fig. 3. **Stark shift above 2 ML NaCl. a** X^- emission spectra for varying tip-substrate separation (left, $V = 2.5\,\mathrm{V}$) and for varying bias voltage (right, $z = 2.7\,\mathrm{nm}$) for a PTCDA-tip on top of 2 ML NaCl. A red shift of the central emission is observed upon reducing z or increasing V. **b** Relative shift of the central emission line position μ extracted from the data in **a**, taking the reference energy μ_0 from the bottom spectrum in **a**. Spectra in **a** are offset vertically for easier comparison. These results are similar to those observed for the PTCDA-tip on top of Ag(111) in Fig. 2d and e. Source data are provided as a Source Data file.

4. Spectra acquisition parameters

Suppl. Table I. Relevant acquisition parameters for spectra presented in Fig. 2 of the main manuscript and Suppl. Fig. 3. All spectra were obtained above clean Ag(111). X^- spectra were acquired with a PTCDA molecule attached to the tip, plasmon spectra with a clean metallic tip.

Fig.	V(V)	I(pA)	t(min)	Spectrum position in the figure
2b	2.5	50	10	top (X^-)
	2.5	50	6	middle
	2.5	50	30	bottom
	2.5	50	2	top (plasmon)
	2.5	1000	30	middle
	2.5	100	6	bottom
2c	2.5	50	6	all X^- spectra
	2.5	50-1000	2-30	all plasmon spectra, different I and t do not influence the plasmon central energy
2d	2.5	7	6	top (z-approach)
	2.5	10	6	
	2.5	20	6	
	2.5	30	6	
	2.5	40	6	
	2.5	50	6	
	2.5	60	6	
	2.5	70	6	bottom
2d	2.9	76	2	top (V-dependency)
	2.8	66	2	
	2.7	56	2	
	2.6	40	2	
	2.5	31	2	bottom
Suppl. Fig. 3a	2.5	1.4	6	top (z-approach)
	2.5	4	6	
	2.5	5	6	
	2.5	7	6	
	2.5	10	6	
	2.5	15	6	
	2.5	20	6	bottom
Suppl. Fig. 3a	2.9	5	6	top (V-dependency)
	2.7	3.8	6	
	2.5	3.2	6	
	2.35	2.6	6	bottom

5. Adatom cluster imaged with a PTCDA functionalized tip

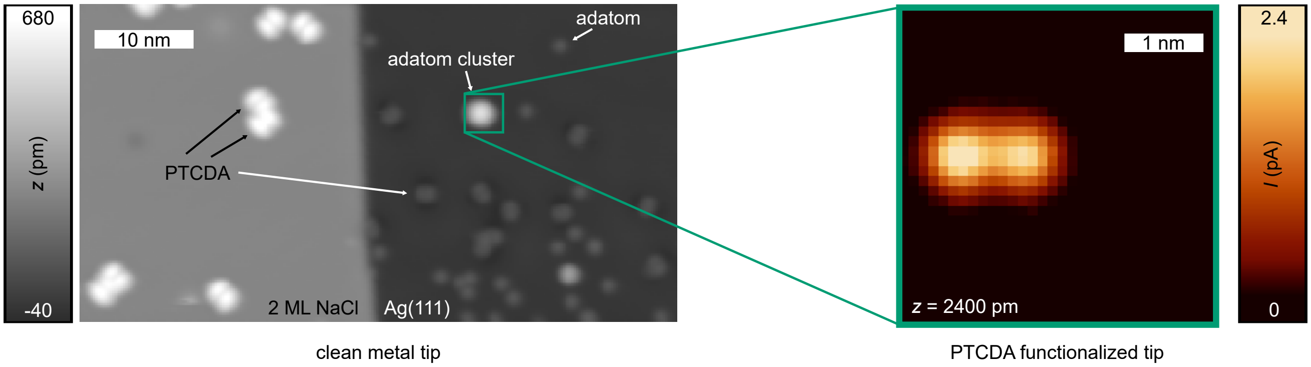

Suppl. Fig. 4. **Constant height current image with PTCDA functionalized tip.** Left: Constant current STM image ($V = -2.5\,\mathrm{V}$, $I = 5\,\mathrm{pA}$) recorded with a clean metal tip showing the clean Ag(111) surface, partially covered with 2 ML NaCl and a random distribution of adsorbed PTCDA molecules. A prominent circular cluster of Ag adatoms is protruding significantly from the Ag(111) surface. Right: After functionalizing the metal tip with a PTCDA molecule the appearance of the adatom cluster changes drastically. The adatom cluster appears as two bright lobes in a constant height STM image ($V = 2.5\,\mathrm{V}$) recorded with the tip retracted to $z \approx 2.4\,\mathrm{nm}$. The image resembles closely a constant height STM image recorded above an upright standing PTCDA molecule [1] and, therefore, confirms the successful functionalization of the tip.

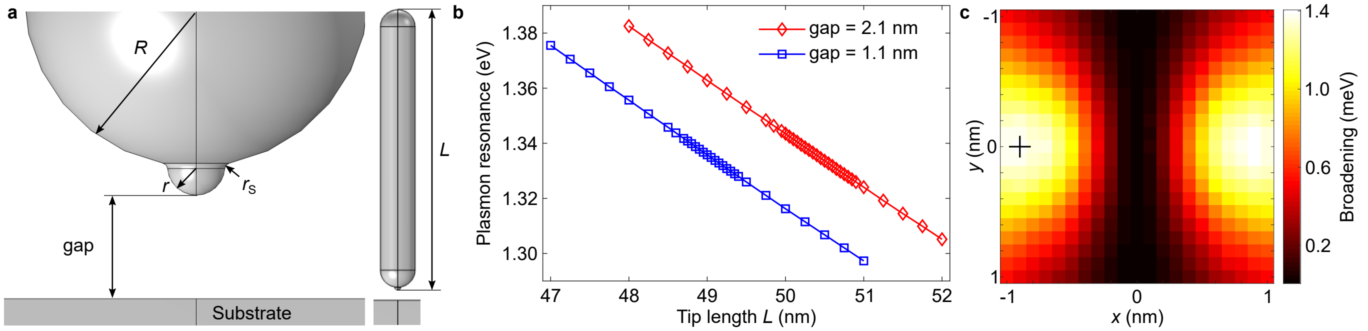

Suppl. Fig. 5. **Exciton decay rates for different tip lengths.** **a** Geometry of the tip and the substrate used to model the plasmonic response. The following parameters were used for the calculation: $R = 3\,\mathrm{nm}$, $r_\mathrm{S} = 0.1\,\mathrm{nm}$, $r = 0.5\,\mathrm{nm}$, gap=2.1 nm (gap=1.1 nm) for the molecule aligned with (perpendicular to) the tip axis. L was varied in the range shown in **b** to tune the plasmon resonance. **b** Plasmon resonance as a function of the tip length for two different gap sizes (gap=2.1 nm - red diamonds, and gap=1.1 nm - blue squares) considered in this work. The position of the plasmon resonance was taken as the position of the maximum of the plasmon-induced broadening for the vertically oriented molecule calculated as a function of the exciton frequency that was varied as a free parameter. **c** Calculated tip-position-dependent map of the line broadening for a molecule positioned perpendicularly to the tip axis in the middle of the gap. The position marked by the black cross is used to calculate the data in Fig. 4e of the main text. Source data are provided as a Source Data file.

Suppl. Note 2. THEORY

To interpret the experimental results, we develop a theoretical model addressing several important aspects of the tunneling process and light emission from PTCDA in an STM junction:

(i) We calculate the tunneling rates between the molecule and the respective electrodes (tip and substrate). To this end, we consider a single electron approximation, where an "active" molecular orbital is isolated within the many-body molecular state, and an electron tunneling between the molecule and the metal is seen as a one-electron energy-conserving transition between this orbital and electronic states of the metal (also termed as resonant electron transfer, RET). The electron dynamics in the system is described with the wave packet propagation (WPP) method [2] yielding the tunneling rates, energies of the "active" molecular orbitals in the STM junction, as well as the spatio-temporal picture of the process.

(ii) We calculate the plasmon-induced decay rate of the molecular excited state (trion) combining the classical electromagnetic description of the tip and the sample with a quantum description of the molecular exciton as described in Ref.[3]. In brief, this model takes the molecular transition charge density (the generalization of molecular transition-dipole moment) obtained from linear-response TDDFT and inserts it as a classical source generating a quasi-static electric field in the dielectric environment formed by the tip and the substrate.

1. (Time-dependent) density-functional-theory calculations

To model the exciton, we perform a linear-response time-dependent density-functional theory (TDDFT) calculation of the PTCDA molecule in a vacuum using Gaussian 16 [4] with the B3LYP hybrid functional [5] and the augmented correlation-consistent valence double zeta basis set AUG-cc-pVDZ. This basis set is augmented by diffuse functions that we include to properly describe the spill-out of the electron density in negative ions. We relax the molecular geometry in its negative ground state D_0^- assuming that the molecule is singly negatively charged when attached to the tip [6, 7]. The ground state of the negative molecule is modelled as a doublet state within the spin-unrestricted Kohn-Sham density functional theory (DFT). From TDDFT we obtain a series of excited states, the lowest of which, denoted D_1^-, appears at the excitation energy of 1.453 eV. We next optimize the molecular geometry in D_1^- and calculate the emission energy of the $\mathrm{D}_1^- \to \mathrm{D}_0^-$ transition $E_{\mathrm{D}_1^- \to \mathrm{D}_0^-} = 1.335\,\mathrm{eV}$, in excellent agreement with the experiment. We also extract the transition charge density ρ of $\mathrm{D}_1^- \to \mathrm{D}_0^-$ shown in Fig. 4d of the main text, which we use as an input for further calculations of the plasmon-induced decay rate.

2. Exciton decay rate

To estimate the plasmon-induced decay rate of the exciton shown in Fig. 4e of the main text, we insert ρ as a source charge density into a finite-element calculation of electrostatic field in the environment defined by the Ag electrodes: the STM tip and the substrate. The geometry of the tip and the substrate is shown in Suppl. Fig. 5 and the relevant parameters are defined in the figure caption. The tip is modelled as a vertically oriented cylindric rod with hemispherical ends, one of which is facing the substrate. At the bottom of this hemispherical cap, there is an additional hemispherical protrusion which mimics the atomic sharpness of the STM tip. The substrate is modelled as a semi-infinite interface. The simulation is performed using the finite-element method implemented in Comsol Multiphysics, version 5.5 [8]. We use the zero-potential condition on the domain boundary and enlarge the simulation domain until convergence is reached.

From the simulation, we extract the quasi-static potential ϕ induced by the environment in response to the source density and estimate the decay rate γ as $\hbar\gamma = 2\mathrm{Im}\{\int \rho\phi \mathrm{d}\mathbf{r}\}$. We next tune the plasmon resonance by adjusting the length of the tip while maintaining the gap of 2.1 nm (1.1 nm) for the configuration of the molecule aligned with (perpendicular to) the tip apex. We probe the resonance by calculating the plasmon-induced broadening as a function of the exciton energy that is treated as a free parameter. The results of this calculation are shown in Fig. 4e (main text), respectively for the molecule aligned with the tip axis and perpendicular to it, for several lengths of the tip.

For the gap size of 2.1 nm, and the center of the vertically oriented molecule positioned in the middle of the gap and the plasmon resonance tuned to the exciton energy, we obtain $\hbar\gamma_{\mathrm{pl}} \approx 24\,\mathrm{meV}$ (see Fig. 4e of the main text). We note that this estimate cannot exactly reproduce the value of the plasmon-enhanced decay of the exciton in the real junction as the exact geometry of the junction is generally not known and other factors, such as radiative damping, can also modify the plasmon response. We therefore treat the value of $\hbar\gamma_{\mathrm{pl}} \approx 24\,\mathrm{meV}$ rather as an order-of-magnitude estimate of the plasmon-enhanced exciton decay rate that we compare to the charge-transfer rate calculated as described in Suppl. Note 2.3.

We also calculate the plasmon-induced decay rate of the molecule in the configuration perpendicular to the tip axis shown in Fig. 4e of the main text as a function of the tip-plasmon resonance. We position the molecule in the middle of the 1.1 nm gap and find the lateral position (x, y) of the tip that maximizes the interaction of the tip plasmon with the molecular exciton. To that end, we first calculate the tip-position-dependent map of the plasmon-induced broadening, shown in Suppl. Fig. 5c, maintaining the 1.1 nm gap and find the tip position for which the broadening is maximal (marked by the black cross in Suppl. Fig. 5c).

3. Charge transfer dynamics

1. The wave packet propagation approach

The WPP approach used here to describe the charge transfer dynamics between the molecule and the substrate was detailed earlier [2]. Therefore, only a brief discussion will be presented here. Because of the coupling with the continuum (metal states), the discrete state (molecular orbital) becomes quasi-stationary reflecting the possibility of an energy-conserving resonant electron transfer between the molecule and the metal. The quasi-stationary molecule-localized state is characterized by its energy and width (charge transfer rate with the substrate, inverse of the lifetime), and it appears as a resonance of the corresponding width in the electron density of states of the system. Within the one-electron picture, the calculation of the energy and width of the molecular resonance considers an electron transfer between the molecular and metallic potential wells. The direction of the electron transfer is set independently by defining the work function of the substrate. Depending on the energy of the molecular orbital active in the resonant charge transfer process with respect to the Fermi level, the molecule/metal charge transfer might correspond to an electron loss or to an electron capture by the molecule (in the latter case one can describe the process as an energy-conserving hole transfer into the metal). Because of the equivalence of the description of the electron or hole dynamics, without loss of generality in what follows we will discuss the electron transfer into the metal.

Within the WPP approach the characteristics of the quasistationary molecule-localized electronic states are obtained from the analysis of the time-evolution of the electron wave function $\psi(\mathbf{r}, t)$ described by the time-dependent Schrödinger equation (atomic units are used unless otherwise stated)

$$i\frac{\partial\psi(\mathbf{r}, t)}{\partial t} = [T + V_{\mathrm{eff}}(\mathbf{r})]\,\psi(\mathbf{r}, t), \tag{1}$$

The initial conditions $\psi(\mathbf{r}, t = 0)$ are given by the orbitals of the free-standing molecule identified as orbitals active in RET. In Suppl. Eq. 1, the kinetic energy operator $T = -\frac{1}{2}\nabla^2$, and $V_{\mathrm{eff}}(\mathbf{r})$ is an effective one-electron potential. It

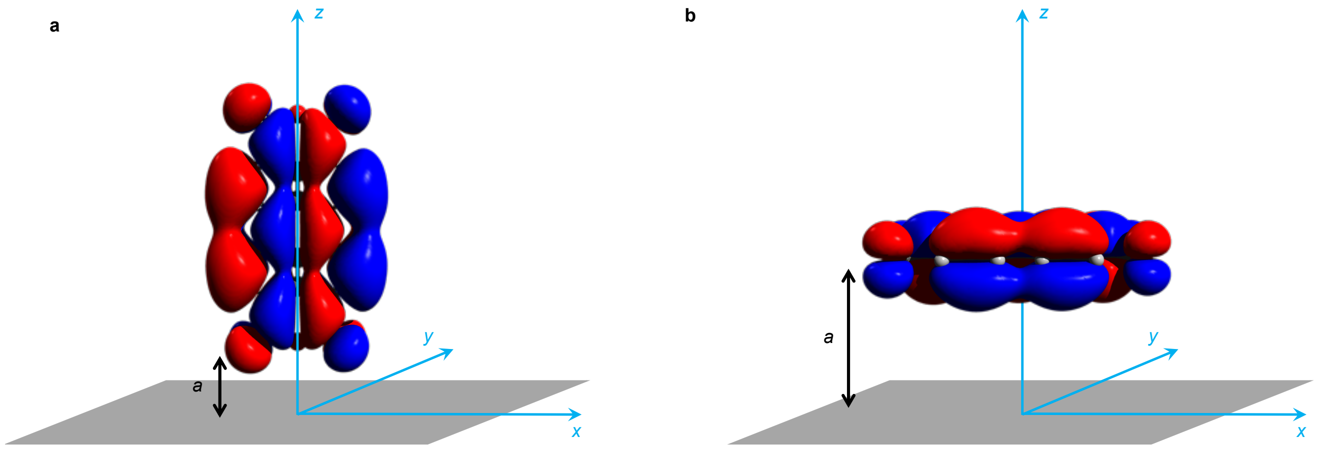

Suppl. Fig. 6. **Molecular geometry used in WPP calculations.** Geometry of the molecule above the metal substrate used in WPP calculations. The molecule represented by the wave function of the LUMO is placed above the metal surface in two configurations: **a** perpendicular to the surface and **b** parallel to the surface. The former configuration mimics the lifted molecule standing upright on the Ag tip. The molecule-surface distance a is measured from the image plane of the metal set as $(x, y, z = 0)$-plane and shaded with gray color.

is defined assuming that the electron-molecule interaction is fully screened inside the metal.

$$V_{\rm eff}(\mathbf{r}) = \begin{cases} V_{\rm mol}(\mathbf{r}) + V_{\rm surf}(z) + \Delta V_{\rm surf}(\mathbf{r}) + V_{\rm abs}(\mathbf{r}), & z \geq z_0, \\ V_{\rm surf}(z) + V_{\rm abs}(\mathbf{r}), & z < z_0. \end{cases} \tag{2}$$

Here z_0 defines the position of the image plane typically located at 2a$_0$ (a$_0$ stands for the Bohr radius) above the surface atomic layer [9]. Without loss of generality, we set $z_0 = 0$ (see Suppl. Fig. 6).

- $V_{\rm mol}(\mathbf{r})$ is the electron-molecule interaction potential. It is given by the sum of local $V^L(\mathbf{r})$ and nonlocal $V^{NL}(\mathbf{r})$ terms $V_{\rm mol}(\mathbf{r}) = V^L(\mathbf{r}) + V^{NL}(\mathbf{r})$.
- $V_{\rm surf}(z)$ is the electron-metal surface interaction potential.
- $\Delta V_{\rm surf}(\mathbf{r})$ stands for the change of $V_{\rm surf}(z)$ induced by the molecule.
- $V_{\rm abs}(\mathbf{r})$ is the optical absorbing potential.

With time-dependent wave function $\psi(\mathbf{r}, t)$ represented on the equidistant 3D Cartesian grid, Suppl. Eq. 1 is solved using the Fourier pseudo-spectral approach [10] and the split-operator technique [11] (the time-propagation step is $dt = 0.02$ a.u.). The 3D Cartesian grid typically comprises $N_x = 768, N_y = 768, N_z = 1280$ nodes and has the same step $h = 0.11$ a$_0$ in x, y, and z-directions. Analysis of $\psi(\mathbf{r}, t)$ yields all the necessary information on the quasi-stationary molecule-localized states in front of the metal surface such as their energies E^j and molecule-substrate charge transfer rates $\gamma^j_{\rm ch}$ (or resonance widths). Here, j labels quasi-stationary molecule-localized states according to the "parent" gas-phase orbital. In practice, we are interested in j =LUMO, LUMO+1, and LUMO+2. The Advantage of the WPP approach is that it allows one to "visualize" the adsorbate/substrate charge transfer process by directly monitoring $\psi(\mathbf{r}, t)$, or by extracting the energy-resolved resonance wave functions $\psi(\mathbf{r}, E^j)$ of decaying molecular orbitals.

$$\psi(\mathbf{r}, E^j) = \int_0^\infty \psi(\mathbf{r}, t) e^{iE^j t} \mathrm{d}t, \tag{3}$$

to this end $\psi(\mathbf{r}, t = 0)$ is set as the corresponding orbital of the free-standing molecule.

Below we detail the components of the effective one-electron potential.

The electron-molecule interaction potential, $V_{\rm mol}(\mathbf{r}) = V^L(\mathbf{r}) + V^{NL}(\mathbf{r})$, is obtained from the ab-initio quantum chemistry Density Functional Theory (DFT) calculations as implemented in the Abinit package [12]. The calculations

are performed within local density approximation with Perdew-Wang correlation energy [13] for the free-standing molecule with the equilibrium geometry given by that of the neutral PTCDA.

Within the one-electron approach to the RET between the molecule and the surface, it is important that the energies of the active orbitals are representative for the energies of the many-body states with respect to the vacuum level and the Fermi energy of the metal as it defines the height of the potential barrier and the direction of the electron transfer ("to" or "from" the metal). Since the molecular anion is formed by an electron attachment to the neutral molecule, the binding energy of the LUMO with respect to the vacuum level should represent the electron affinity of PTCDA (3.25 eV [14], 3.07 eV [15]). However, since Koopmans' theorem [16] does not apply for the DFT case, the binding energy of the LUMO as obtained with Abinit is appreciably different from the molecular affinity. Using conclusions of the study performed with hybrid functionals [14], we then defined the $V_{\rm mol}(\mathbf{r})$ potential as follows.

$$V_{\rm mol}(\mathbf{r}) = \underbrace{\zeta V^{L}_{\rm PTCDA}(\mathbf{r}) + (1-\zeta)\; V^{L}_{\rm PTCDA^-}(\mathbf{r})}_{V^L(\mathbf{r})} + V^{NL}(\mathbf{r}), \tag{4}$$

where $V^{L}_{\rm PTCDA}(\mathbf{r})$ is the local potential (the sum of Hartree and exchange-correlation contributions) calculated for the free-standing neutral molecule, and $V^{L}_{\rm PTCDA^-}(\mathbf{r})$ is the local potential calculated for the free-standing molecular anion assuming 1/2 occupation of the frontier orbitals of both spins. The nonlocal potential is given by the norm-conserving pseudopotentials in the Kleynman Bylander form [17] as implemented in Abinit to describe the electron interaction with individual atoms forming the molecule. For $\zeta = 0.32$ from the WPP calculations for the free-standing molecule, we obtain the energy of the LUMO $E_{\rm LUMO} = -3.19$ eV with respect to the vacuum level, i.e., in good agreement with PTCDA affinity reported earlier [14, 15]. Simultaneously $E_{\rm LUMO+1} = -1.88$ eV and $E_{\rm LUMO+2} = -1.85$ eV so that $E_{\rm LUMO+2} - E_{\rm LUMO} = 1.34$ eV close to the trion fluorescence X^- peak energies observed in our experiments.

The electron-metal surface interaction $V_{\rm surf}(z)$ is represented by the model potential of Jennings et al [18].

$$V_{\rm surf}(z) = \begin{cases} -\frac{1}{4(z-z_0)}\left\{1 - e^{-\alpha_{\rm J}(z-z_0)}\right\}, & z > z_0, \\ -\frac{V_0}{A_{\rm J}e^{B_{\rm J}(z-z_0)}+1}, & \text{otherwise}, \end{cases} \tag{5}$$

where $A_{\rm J} = 4V_0/\alpha_{\rm J} - 1$, $B_{\rm J} = 2V_0/\alpha_{\rm J}$. As follows from the equation above, $V_{\rm surf}(z)$ is only a function of the electron coordinate z perpendicular to the surface. It smoothly joins the classical image potential $-1/4(z - z_0)$ for an electron being in a vacuum ($z \gg z_0$) with a constant potential $-V_0$ inside metal. We have also performed the calculations with the z-dependent model potential developed by Chulkov and collaborators [9] to describe an electron interaction with Ag(111) metal surface corresponding to the substrate. In this case the band structure of Ag(111) along the direction perpendicular to the surface is well reproduced (projected band gap, surface and image potential states). Consistent with earlier results [2, 19], the projected band structure shows only mild effect on the electron transfer process. Considering this result as well as the experimental procedure where the STM tip is covered with a Ag layer by indentation of the tip into Ag surface so that the structure of the Ag layer is ill-defined, we decided to use the jellium description of the metal to characterise the electron transfer with both: the tip and the substrate. The potential parameters are set as $\alpha_{\rm J} = 1.1715$ a.u., $V_0 = 12$ eV $= 0.4410$ a.u. consistent with the potential tail at metal/vacuum interface and the valence band bottom of silver [9].

The change of the electron-metal interaction because of the presence of the molecule $\Delta V_{\rm surf}(\mathbf{r})$ is set as

$$\Delta V_{\rm surf}(\mathbf{r}) = \begin{cases} -V^L(x, y, -(z+a)), & z > 0. \\ 0, & \text{otherwise} \end{cases} \tag{6}$$

This choice guarantees that at the image potential plane defined with $\mathbf{r}_{\rm IP} = (x, y, 0)$ the local part of the electron-molecule interaction is fully screened $V^L(\mathbf{r}_{\rm IP}) + \Delta V_{\rm surf}(\mathbf{r}_{\rm IP}) = 0$. Since the non-local potential $V^{NL}(\mathbf{r})$ consists of very short-range contributions around the atoms constituting the molecule (nonlocal potential range smaller than molecule-surface distance a), we also obtain that $V_{\rm mol}(\mathbf{r}_{\rm IP}) + \Delta V_{\rm surf}(\mathbf{r}_{\rm IP}) = 0$. The electron-molecule interaction is smoothly screened at the metal surface.

The optical absorbing potential $V_{\rm abs}(\mathbf{r})$ is introduced at the boundaries of the computational box to impose the outgoing wave boundary conditions[20, 21] consistent with the search for the quasi-stationary molecule-localized states coupled with the continuum of the electronic states of the metal. For the explicit form of $V_{\rm abs}(\mathbf{r})$ see Ref. 2.

4. Analysis of the charge transfer process

To provide further insight into the dynamics of charge transfer from the molecule to the metal surface we analyse in Suppl. Fig. 7 the wave functions of molecular resonances defined with Suppl. Eq. 3. In particular, we investigate

the probability distribution associated with LUMO (Suppl. Fig. 7a,d), LUMO+1 (Suppl. Fig. 7b,e), and LUMO+2 (Suppl. Fig. 7c,f). We consider both the upright configuration of the molecule (Suppl. Fig. 7a-c), and the flat-lying configuration (Suppl. Fig. 7d-f). The probability density $\ln(|\psi(\mathbf{r}, E^j)|^2)$ is shown in Suppl. Fig. 7 in the plane perpendicular to the metal surface. The dashed line defines the image potential plane of the metal located at $z = 0$. It sets the metal ($z < 0$) / vacuum ($z > 0$) interface.

In the vacuum region one recognizes the electron density of the corresponding molecular orbitals. Inside the metal the probability density is leaking into the metal bulk reflecting the outgoing electron flux because of the population decay of molecular resonance. While the charge transfer is most efficient along the direction perpendicular to the surface (lowest tunneling barrier), the exact pattern of the decay depends on the nodal structure and symmetry of the "parent" molecular orbital. Notice that for the LUMO+1 and LUMO+2 states located above the Fermi level of the tip, the electron is transferred from the molecule to the metal, while for LUMO orbital the wave packet analysis reflects the hole transfer to the metal.

From the comparison of the upright configuration and the flat-lying configuration it is also clear that the contact area between the molecule and the substrate strongly determines the degree of hybridization of the molecular orbital with the continuum of states in the metal. Overall, the metal continuum part of the resonant wave functions is more pronounced for the flat-lying configuration reflecting faster charge transfer rates (see Fig. 4 of the main text).

5. Influence of the protrusion geometry on the plasmon-induced broadening

To assess the influence of the exact detailed shape of the tip protrusion on the plasmon-exciton interaction we have evaluated the plasmon-induced broadening for a series of geometries. Concretely, we have used a single protrusion of a radius varying between r_{p} =0.2 nm and 0.5 nm, and a double protrusion composed of a pair of overlapping hemispheres as shown in Suppl. Fig. 8a,b. The molecule was placed in the middle of the gap in the vertical orientation and the gap was held constant for all protrusion geometries. Since the resonance frequency of the plasmon varies slightly when different protrusions are considered (Suppl. Fig. 8c,d), we pick the maximum of the calculated plasmon-enhanced broadening for each protrusion geometry and compare these maxima in Suppl. Fig. 8e. We note that the slight frequency shift could in principle be compensated by adjusting the geometry (total length) of the model tip and therefore does not play a significant role. For reference, we also plot the position of the plasmon resonance for each geometry in Suppl. Fig. 8f. The Purcell effect (plasmon-enhanced broadening) was calculated as in Fig. 4e of the main text. The results calculated with the double protrusion show only a minor deviation from the data where a single-atom protrusion was considered. It therefore appears that the precise nature of the protrusion does not play a significant role.

6. Vibronic features in the emission spectrum

In the experimentally observed spectra a vibronic progression can be observed [22–24]. To assign the vibronic peaks we calculate a vibronic emission spectrum of a PTCDA molecule in a vacuum using the FCHT module implemented in Gaussian 16 rev. C.01 and using the B3LYP/AUG-cc-pVDZ level of theory at T=0 K. When plotting the spectrum we include an artificial broadening of $10\,\mathrm{cm}^{-1}$. In Suppl. Fig. 9 we compare the calculated spectrum (black line) with an experimentally obtained spectrum (red line) of a PTCDA molecule adsorbed flat on 3 ML NaCl. The spectrum corresponds to the data presented in Fig. 2a of the main manuscript. The calculated spectrum shows that the zero-phonon line (ZPL) is dominating the spectrum as the vibronic activity in PTCDA is relatively low. This finding confirms our assignment of the most intense peak as the ZPL of the molecular emission. In the inset we also show the calculated vibrational modes that correspond to the most intense peaks of the vibronic shoulder that can be straightforwardly assigned to the experimental peaks. The vibronic peaks appearing at $-231\,\mathrm{cm}^{-1}$ and $+238\,\mathrm{cm}^{-1}$ are those observed in the spectra of the lifted PTCDA, and can be assigned to the in-plane breathing mode of the molecule. The peaks of lower energy ($56\,\mathrm{cm}^{-1}$ and $129\,\mathrm{cm}^{-1}$) are not present in the calculated spectrum and can be attributed to vibrational modes that arise from the interaction of the molecule with the substrate, as discussed in Ref. [22].

We note that the experimental spectrum features vibronic peaks on the high energy side of the ZPL and we attribute them to the hot emission emerging from a vibrationally excited molecule. The exact origin of the vibrational pumping mechanism leading to hot luminescence may originate from inelastic electron tunneling as well as being related to the sequential charging of the molecule and subsequent photon emission through e.g. the Franck-Condon mechanism. Unraveling the exact details of this process is beyond the scope of our manuscript.

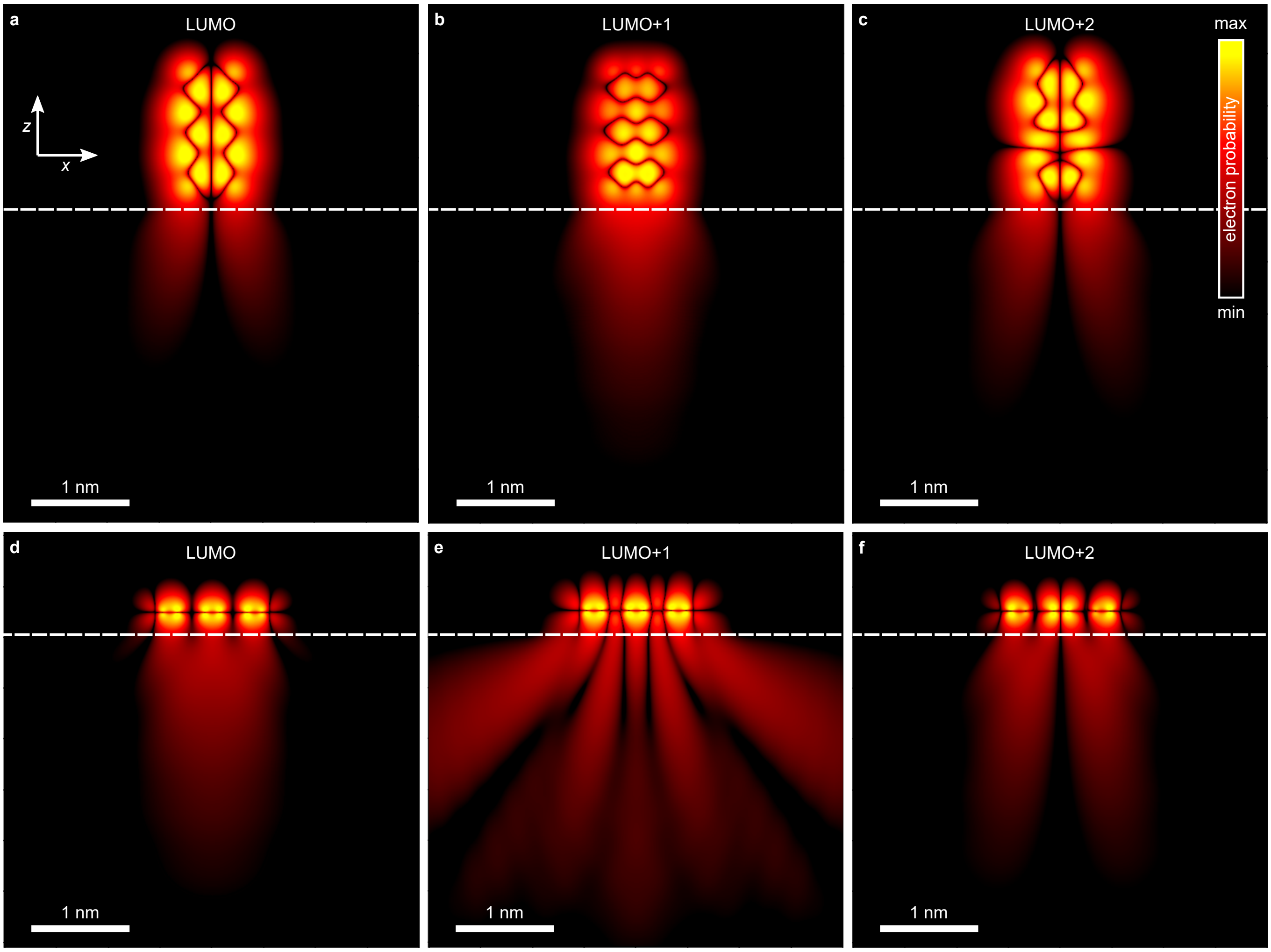

Suppl. Fig. 7. **Orbital leaking into jellium substrate.** Electron probability distribution at orbital resonances calculated for a PTCDA molecule **a-c** standing upright on a jellium Ag surface, and **d-f** lying flat on the metal substrate, shown for **a**, **d** LUMO, **b**, **e** LUMO+1 and **c**, **f** LUMO+2. At the start of the propagation, the wave function has the form of the respective orbital of the unperturbed molecule. The molecule is at a distance of $5\,\mathrm{a}_0$ from the image plane of the surface marked by the dashed line. The color represents the square modulus of the wave function which is displayed $y = 2.5\,\mathrm{a}_0$ above the $y = 0$ plane, which is chosen to be a symmetry plane of the molecule. In all cases the dynamical range of the color scale is e^{-12} to 1. The color scale applies to all panels. Source data are provided as a Source Data file.

7. Influence of tilting of the molecule on calculated rates

Since the exact adsorption geometry of the molecule on the tip is not precisely known and even on the planar surface the molecule is held upright only by weak dispersive forces, we consider here the possibility that the molecule is not adsorbed in a perfectly upright configuration (the molecule is tilted). We assume two possible tilting geometries. First we considered that the molecule pivots around an axis perpendicular to the molecular plane that passes through one of the lateral oxygen atoms (Suppl. Fig. 10b,d). In the second configuration the molecule was rotated around the axis passing through the two lateral oxygen atoms (Suppl. Fig. 10c,e). We calculate the plasmon-induced decay rate γ_{pl} considering the same tip-substrate geometry as in Fig. 4 of the main text. For the calculation of the charge-transfer rate γ_{ch} from LUMO of the molecule we consider that in the upright configuration (0°) the molecule is $5\,a_0$ (a_0 being the Bohr radius) from the image plane of the jellium surface. The resulting rates are plotted as a function of the tilting angle in Suppl. Fig. 10a. γ_{pl} is shown as red (black) diamonds corresponding to the tilting scenario in Suppl. Fig. 10b (Suppl. Fig. 10c) and for both considered geometries presents a steady decrease of its value from $\approx 24\,\mathrm{meV}$ roughly following a cosine-like behaviour. The red (black) circles mark γ_{ch} calculated using the tilting geometry shown in Suppl. Fig. 10d (Suppl. Fig. 10e). For the geometry considered in Suppl. Fig. 10e γ_{ch} grows steadily and for

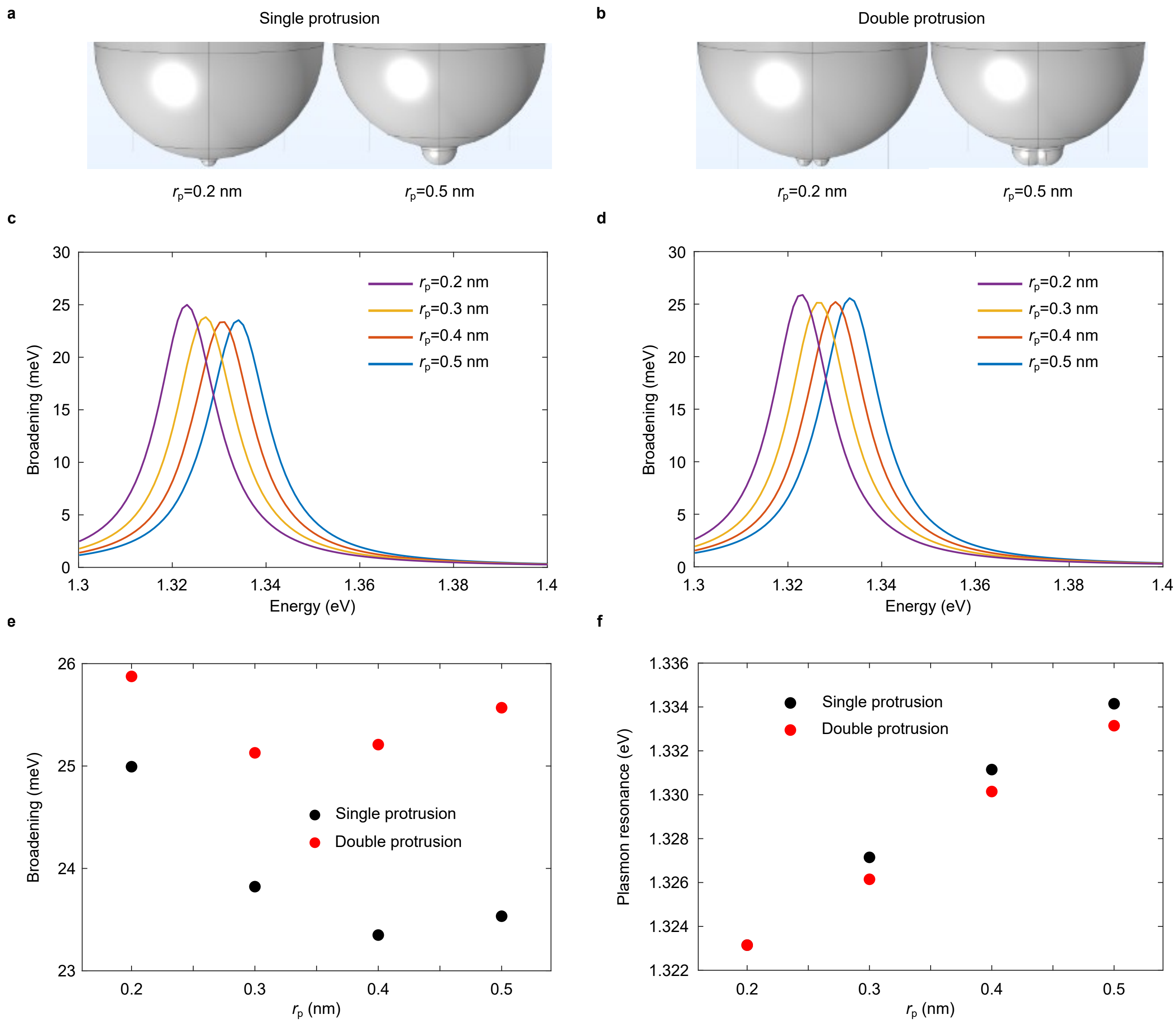

Suppl. Fig. 8. **The influence of the picocavity shape on the plasmon-enhanced decay.** **a**, **b** Two picocavity shapes have been taken into account: **a** single hemispherical protrusion, and **b** double protrusion. The radius r_p of these protrusions is varied and for each geometry the spectrum of the plasmon-induced trion broadening. **c**, **d** the spectra corresponding to the geometries shown in **a**, **b**. **e** Comparison of the calculated maxima of the spectra in **c**, **d**. **f** Comparison of the position of the plasmon resonance when considering different picocavity geometries. Source data are provided as a Source Data file.

the angle of 45° reaches almost the calculated values of γ_pl. This suggests that exciton quenching could be expected for significant tilting angles. On the other hand, for the geometry of Suppl. Fig. 10d γ_ch first decreases, reflecting the initially decreased contact between the molecule and the surface, and then slowly varies without significantly increasing in value. We thus conclude that, for this geometry, exciton quenching due to the charge-transfer processes would be less likely.

Overall, we believe that our qualitative conclusions on the respective role of the electronic and electromagnetic channels in the trion decay as follows from the results shown in Fig. 4 of the main text are robust with respect to the possible molecular tilt.

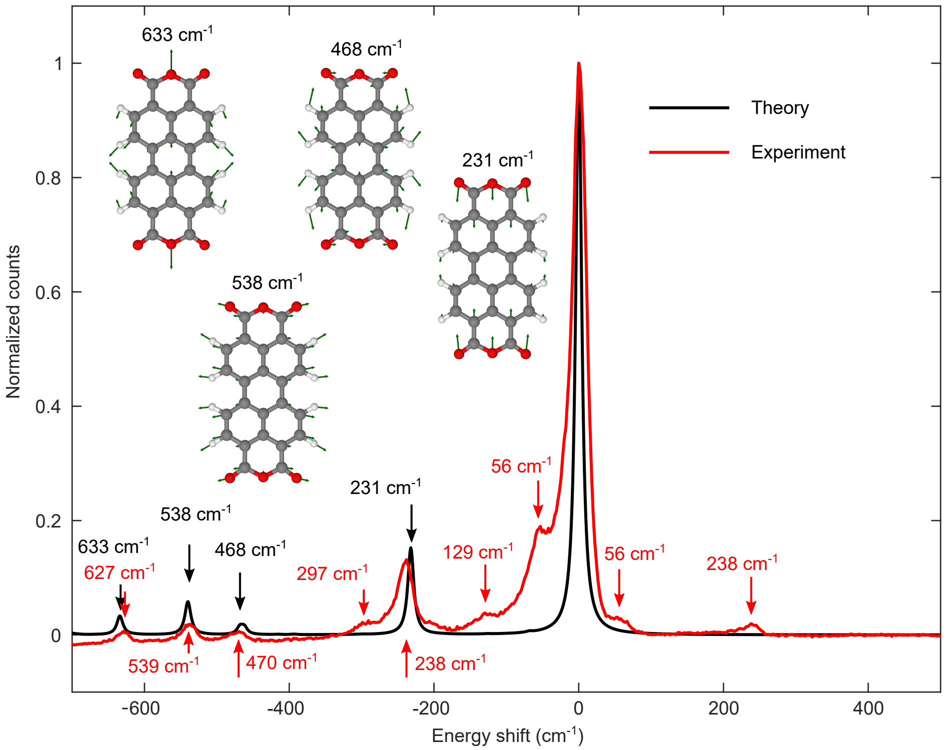

Suppl. Fig. 9. **Vibronic emission spectrum.** Photon emission spectrum obtained experimentally (red line) and theoretically (black line) calculated for zero temperature. The most intense vibronic peaks are labelled with their resonance energy. The black labels correspond to the vibrational frequencies calculated for a molecule in a vacuum using Gaussian 16, the red labels correspond to the experimental positions of the peaks. The energy scale is defined relative to the ZPL energy. Acquisition parameters for the experimental spectrum: $V = -2.5\,\mathrm{V}$, $I = 60\,\mathrm{pA}$, $t = 30\,\mathrm{min}$. Source data are provided as a Source Data file.

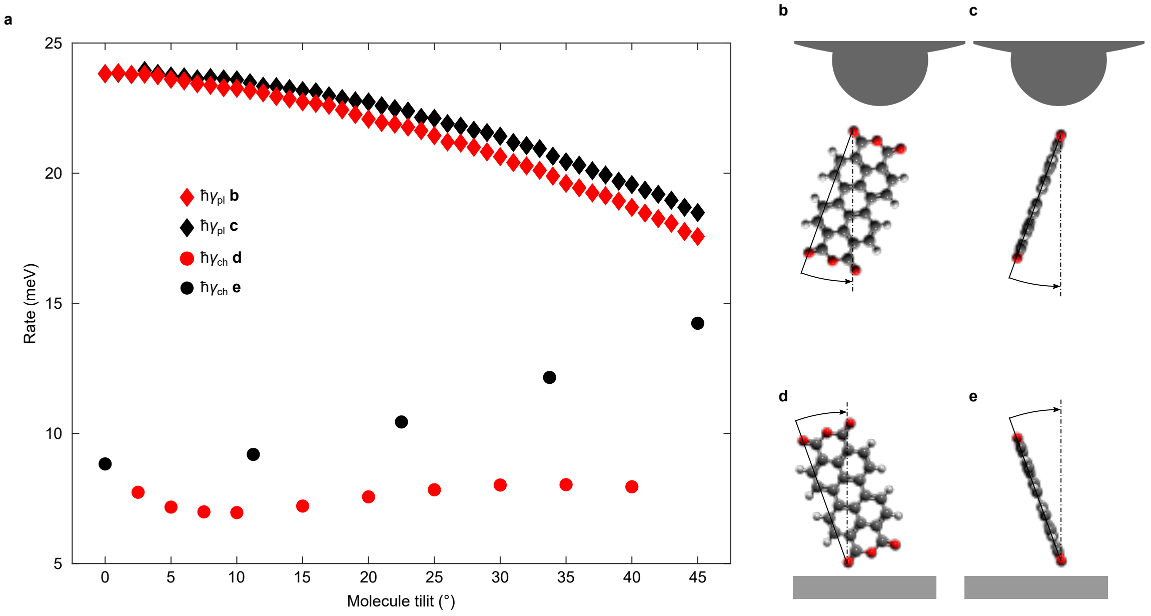

Suppl. Fig. 10. **Decay rates for different tilt angles.** Plasmon-induced decay rate γ_{pl} and charge-transfer rate γ_{ch} of LUMO calculated as a function of the molecule tilt angle for the geometries shown in **b-e**. Source data are provided as a Source Data file.

8. Excitation mechanisms of suspended molecules

In the main text, we briefly discuss the transport mechanism that drives the suspended PTCDA molecule in the excited state. Here, we provide some more details on these mechanisms, especially for cases where the suspended molecule is facing (i) a bare Ag(111) substrate and (ii) an Ag(111) substrate covered with 2 ML of NaCl. In Suppl. Fig. 11a (top) we schematically show the geometry of the molecule suspended in the gap, and (bottom) a simplified band diagram of the Ag electrodes, the tip and the substrate, showing aligned work functions in the absence of external bias. In Suppl. Fig. 11c we show a many-body energy diagram of a PTCDA-tip facing a bare Ag(111). In this diagram we show the energies of the most relevant states that enter the charge transfer dynamics: The ground (excited) doublet state of the negative molecule D_0^- (D_1^-), the neutral singlet ground state S_0^0, the neutral triplet state T_1^0, and the singlet ground state S_0^{2-} of the doubly negative molecule. In the insets, we also schematically show the dominant electron configurations representing the respective states. Since the molecule is in electrical contact with the tip, we align the many-body levels with respect to the work function of the tip, as opposed to the representation where the levels are aligned with respect to the vacuum level. In this representation, it is therefore assumed that the molecule can exchange an electron with the tip and that this electron is at the Fermi level of the metal. Here, the lowest-lying state is naturally stabilized by the charge transfer with the tip.

The situation is somewhat different when the molecule is suspended in a gap between the tip and a NaCl/Ag(111) substrate as shown in Suppl. Fig. 11b (top). The NaCl layer is schematically depicted in the single-particle diagram in Suppl. Fig. 11b (bottom) via the green rectangle marking the gap between the valence band maximum - VBM - and the conduction band minimum - CBM. The work function of the NaCl/Ag(111) interface is significantly lowered compared to the Ag(111) surface. Since the Fermi levels of the tip and the substrate align at zero external bias, the difference of 1 eV between the vacuum level on the tip and the substrate side gives rise to a static electric field across the gap, which acts on the levels of the suspended molecule. This static field affects the level diagram of the suspended molecule as shown in Suppl. Fig. 11d. Here, the grey dashed lines mark the original positions of the levels shown in Suppl. Fig. 11c. These are shifted by the static electric field (expressed here as an effective bias offset of $V_0 = 1\,\mathrm{V}$) towards higher energies for the neutral states, and to the lower energy for the doubly negative state. We assume that $\alpha = 1/3$ of the voltage drop occurs on the tip side, which we approximately derive from the relative position of the molecule within the gap and which is consistent with the position of the tunneling thresholds in the $\mathrm{d}I/\mathrm{d}V$ spectra.

We now examine what happens when an external bias is applied in the simpler case of a molecule facing the Ag(111) substrate. Here, the Fermi levels of the metal electrodes are no longer aligned and the many-body diagram of the PTCDA states depends on the electrode with which the molecule exchanges electrons. Moreover, the additional voltage drop between the tip and the molecule further modifies the alignment of the levels. In Suppl. Fig. 11e (top), we show such level diagrams drawn for PTCDA on top of the bare Ag(111). The black frame shows how the diagram changes upon the application of a voltage $V = 2.4\,\mathrm{V}$, assuming that the molecule exchanges electrons with the tip. In contrast, the red frame shows the diagram assuming the same voltage conditions, but for electrons exchanged with the substrate. As the voltage drops on both sides of the molecule are different, this results in a different alignment of the many-body levels. A simple rule can be derived from the energetic ordering of the levels: a spontaneous exchange of an electron with the tip (the substrate) can occur only if the energy of the initial many-body state in the respective diagram is higher than the energy of the final one. This rule stems from the fact that an electron escaping the molecule must propagate into an unoccupied state of the metal above the Fermi level, and conversely, an electron captured by the molecule can only originate from the occupied states of the metal below the Fermi level. For the threshold bias of about 2.4 V, we see that the molecule is stabilized by the efficient tip-mediated charge transfer in the state D_0^-, as can be seen in the diagram with the black frame. D_0^- can with a smaller tunneling probability decay into S_0^0 and T_1^0 by releasing an electron into the substrate as shown in the red frame. On the occasion that T_1^0 is populated, the tip can rapidly provide an electron and bring the molecule to the D_1^- excited state (black frame). This state can then radiatively decay back to the negative ground state D_0^-. These features are manifested in the $\mathrm{d}I/\mathrm{d}V$ spectra and corresponding photon-emission thresholds shown in Suppl. Fig. 11e (bottom). At the bias voltage of about 2.3 V, we observe a smooth step in the $\mathrm{d}I/\mathrm{d}V$ curve, corresponding to the $\mathrm{D}_0^- \to \mathrm{T}_1^0$ transition, accompanied by a gradual onset of photon emission. The smooth onset of this process can be understood as a result of voltage-dependent tunneling rate between the molecule and the tip, a result of the modification of the tunneling barrier with increasing voltage.

In contrast, for the molecule suspended above the 2 ML NaCl/Ag(111) substrate, we observe in the $\mathrm{d}I/\mathrm{d}V$ a sharp peak at $\approx 2.35\,\mathrm{V}$ accompanied by a sharp onset of photon emission, as shown in Suppl. Fig. 11f (bottom). To understand this behavior, we plot in Suppl. Fig. 11f (top) the corresponding level diagrams at the bias of about 2.4 V, roughly corresponding to the onset of the experimental features, aligned with respect to the tip (black box) and the substrate (red box). Because of the reduced barrier height on top of NaCl and the associated bias offset of the molecular energy levels, at $\approx 2.35\,\mathrm{V}$ the molecule is stabilized in the doubly negative charge state S_0^{2-} (black box). The sharp $\mathrm{d}I/\mathrm{d}V$ feature in Suppl. Fig. 11f (bottom) is consistent with such a charging event. From then, a substrate-mediated channel to D_1^- opens (red box), eventually leading to the emission of the molecule suspended at

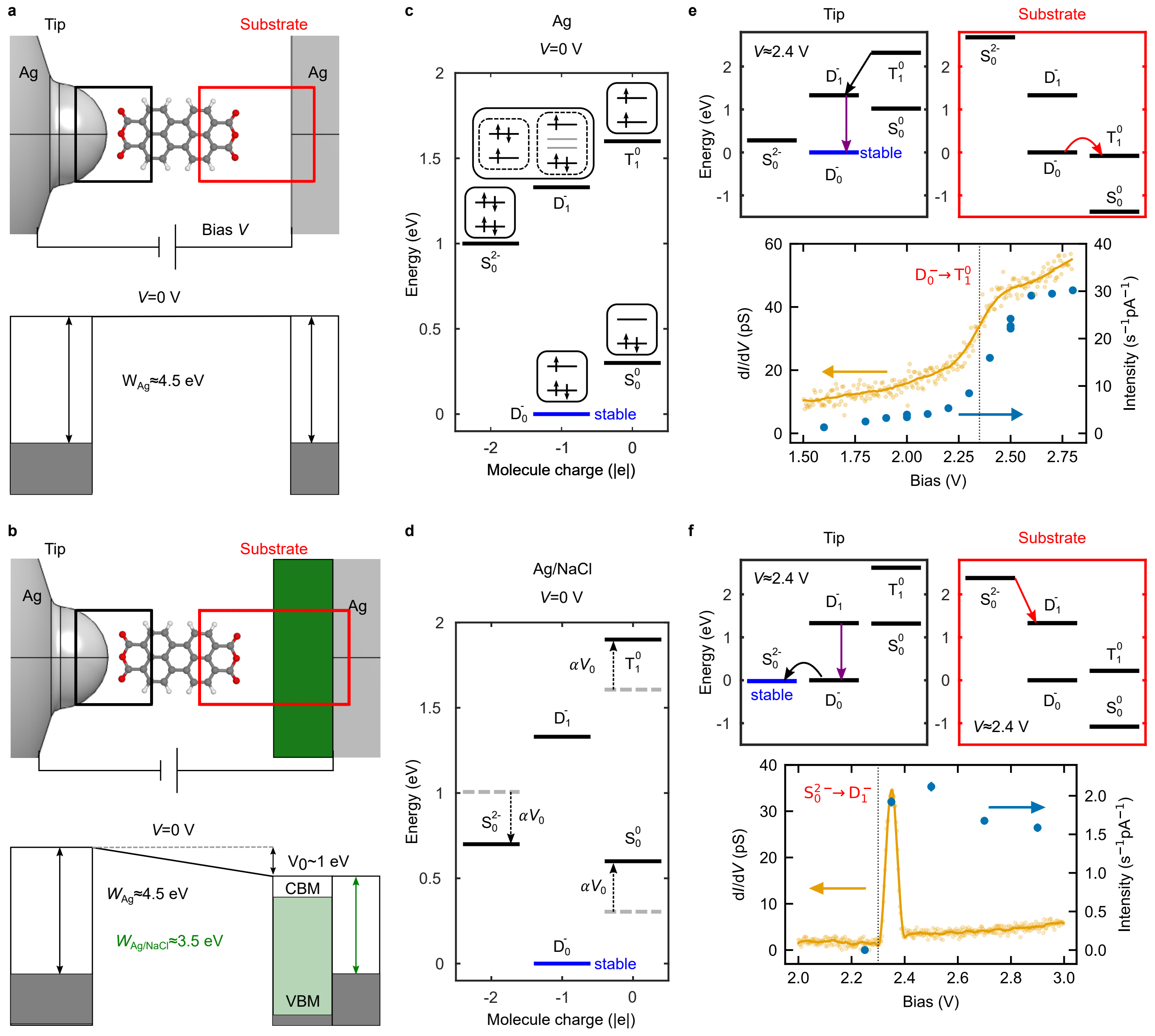

Suppl. Fig. 11. **Exciton creation mechanism.** Schematic representation of the geometry of the molecule suspended on the tip and facing the **a** Ag(111) and **b** NaCl/Ag(111) surface. Alongside with the geometry we show schematic single-particle band diagrams characterising the tip and the substrate. In the diagram we mark the work functions W_{Ag} and $W_{\mathrm{Ag/NaCl}}$ as well as the offset voltage drop V_0 caused by the difference of the tip and the substrate work functions, and the conduction-band minimum (CBM) and valence-band maximum (VBM) of NaCl. In **c**, **d** we show many-body energy diagrams of the molecule where the charge is transferred from/to the Fermi level of the tip. The Ag substrate is considered in **c**, and the NaCl/Ag in **d**. The state stabilized by the tip is drawn in blue and marked as stable. **e**, **f** $\mathrm{d}I/\mathrm{d}V$ curves, corresponding photon intensity, and many-body level diagrams drawn at the threshold voltage. In the black frame we show the diagrams assuming charge-exchange with the tip, and in the red frame the diagrams assuming charge exchange with the substrate. The purple arrows represent exciton decay (mediated by the tip plasmon). Source data are provided as a Source Data file.

the tip. In this example, the D_1^- state is thus populated by a different mechanism than for the molecule-tip in front of the bare Ag(111).

9. Effects of dielectric environment

In the main text we discuss that when the molecule-functionalized tip is placed on top of 2 ML NaCl/Ag(111) the molecular emission line shifts towards red by $\approx 21\,\mathrm{meV}$ compared to the configuration when the molecule is lifted above the bare Ag(111) surface. We show that this shift is consistent with the Stark shift caused by the change in the work function of the substrate. However, other effects related to the change in the dielectric environment of the molecule can be at play as well. Here we discuss three prominent effects that may be encountered when the molecule is moved to a different dielectric environment in the context of STM: the photonic Lamb shift [3] (dynamical screening), the static Stark effect (discussed in the main text), and the static dielectric screening of the molecular charge.

First, we estimate the value of the Lamb shift that results from the inclusion of a thin insulating layer of NaCl. The Lamb shift can be defined as $\hbar E_{\mathrm{Lamb}} = \mathrm{Re}\{\int \rho\phi \mathrm{d}\mathbf{r}\}$ [3], where ρ is the transition density of the molecule and ϕ is the dynamical potential induced by the dielectric environment. Due to the low refractive index of NaCl at optical frequencies (we stress here the difference between the static and dynamical screening), the effect of including the layer primarily manifests itself by an increased distance of the molecule from the Ag substrate. We have therefore evaluated the Lamb shift for a range of molecule separations from the metal, z (z is defined as the distance of the center of the molecule from Ag surface - relevant for the change of the distance between the molecule-functionalized tip and the metal surface upon moving the tip from the clean metal surface to an adsorbed NaCl island). The data is offset such that the value of the Lamb shift is zero at $z = 1.05\,\mathrm{nm}$. To that end we used (i) an approximation where the molecule is treated as a point dipole (black diamonds in Suppl. Fig. 12) and the tip is not considered, and (ii) a more complete model where both the tip and the Ag substrate are present and the molecule is represented by its dynamically oscillating transition density (red diamonds). In the latter case we maintained the distance between the molecule and the tip as well as the length of the tip while increasing the gap. This resulted in a shift of the tip-substrate plasmon resonance. To avoid artefacts associated with the plasmon resonance energy shift, we have therefore evaluated the Lamb shift at the energy corresponding to this plasmon resonance. In both cases we see that the Lamb shift variation with changing the molecule-substrate distance, reaching units of meV, is not sufficient to explain the $\approx 20\,\mathrm{meV}$ shift observed experimentally. The experimental value would correspond to the difference between the Lamb shift evaluated for a smaller distance (closer to the Ag surface) and a value evaluated at a larger distance (i.e., the distance to the Ag surface offset by additional $\approx 0.5\,\mathrm{nm}$ due to the presence of 2 ML NaCl, which is evaluated from the experimental apparent height of the layer). Moreover, we see that the Lamb shift towards the red would be reduced when the molecule is on NaCl, which would lead to the blue shift of the molecular resonance on NaCl, i.e., to the opposite qualitative behaviour compared to the experiment.

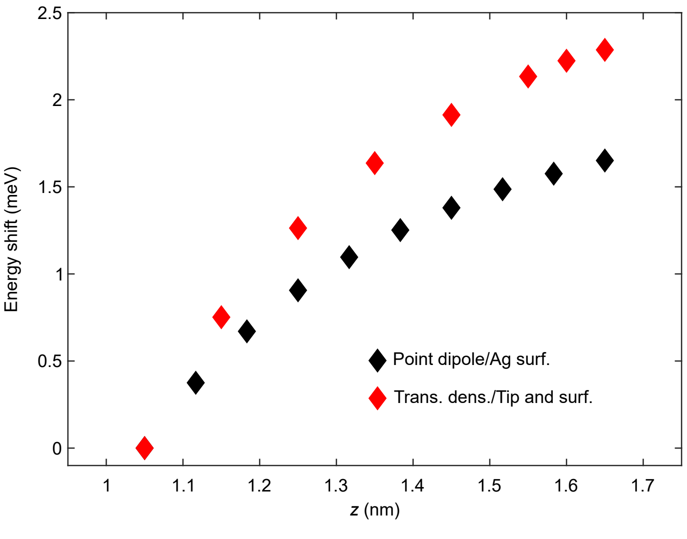

Suppl. Fig. 12. **Lamb shift for different cavity sizes.** Values of the Lamb shift calculated as a function of the distance z of the center of the molecule from the Ag surface. The Lamb shift was offset such that it is zero for the distance of $z = 1.05\,\mathrm{nm}$. The calculation was done using a point-dipole approximation for the molecule and not considering the tip (black diamonds), and considering the transition density and including the tip (red diamonds). In the latter case the tip-molecule distance was held constant and the value of the Lamb shift was evaluated at the resonance of the plasmon to avoid spurious effects due to the fact that the plasmon resonance is shifting with increasing z. Source data are provided as a Source Data file.

The static Stark effect is caused by the action of the static electric field present in the environment on the difference Δp between the static dipole moment of the molecule in the ground and excited state, respectively. In the experiment,

the molecular symmetry is broken due to its adsorption on the tip. This symmetry breaking is expected to induce a static dipole moment in the molecule although the molecular symmetry in a vacuum would suggest its absence. Δp can be estimated using the data derived from the Stark shift experiment (Fig. 2e). We estimate $\Delta p \equiv c_{\rm S} = 0.06\,{\rm e\,nm}$ - as discussed in the manuscript. The static Stark shift is an important first-order phenomenon and we can estimate it experimentally via the bias and distance-dependent measurements presented in the main text. The estimated 30 meV shift is consistent with the experimentally observed 21 meV shift of the emission line that can be associated with the voltage drop in the gap induced by the presence of the NaCl layer (associated with the change in the substrate work function).

Finally, we address the effect of the induced static screening charges on the excitation energy. In the leading order, it would result from the interaction of Δp with the static screening charges in the dielectric caused by the static charge of the molecule (since the molecule is negatively charged). Since both Ag and NaCl perfectly screen the static charge, the difference in energy shift between the molecule-on-NaCl and molecule-on-Ag configuration comes from the geometrical distance z of the molecule from the Ag/vacuum or NaCl/vacuum interface (and its variation Δz between the two configurations). We estimate the value of this induced shift by considering the interaction between the charge in the dielectric, induced by the molecular interaction, with the static dipole moment of the molecule, as:

$$\Delta E_{\rm ind} = \frac{e c_{\rm S}}{8\pi\varepsilon_0 z^3}\Delta z, \tag{7}$$

where ε_0 is the vacuum permittivity and e the elementary charge. We insert $z = 1\,{\rm nm}$ for the effective distance of the center of the molecule to the interface and $\Delta z = -0.05\,{\rm nm}$ its variation when the molecule-functionalized tip is moved from Ag surface to the NaCl island. The latter is estimated from the experimentally recorded position of the tip and the thickness of 2 ML of NaCl. Using these values we estimate that the difference between the shift induced on NaCl and on Ag is about $\Delta E_{\rm ind} \approx -2.2\,{\rm meV}$. That is, the effect is one order of magnitude smaller than the estimation of the Stark shift and the experimental observation.

We thus conclude that out of the three effects considered, the static Stark shift is the strongly dominant contribution in the experimentally observed shift of $-21\,{\rm meV}$. However, since the molecule is brought to the excited state by dynamical charging and, as we discuss in the Suppl. Note 2.8, this mechanism may differ between the configuration when the molecule is on NaCl/Ag and on Ag, we cannot exclude additional effects related to the dynamical charging mechanism. In general, we believe that the interaction of the molecular excitation with the complex environment of the STM should become the focus of future theoretical and experimental efforts that may help unambiguously resolve the origin of the spectral shifts observed in our experiment.

---

[1] T. Esat, M. Ternes, R. Temirov, and F. S. Tautz, Electron spin secluded inside a bottom-up assembled standing metal-molecule nanostructure, Physical Review Research **5**, 033200 (2023).

[2] F. Aguilar-Galindo, A. G. Borisov, and S. Díaz-Tendero, Ultrafast dynamics of electronic resonances in molecules adsorbed on, J. Chem. Theory Comput. **17**, 639 (2021).

[3] A. Rosławska, T. Neuman, B. Doppagne, A. G. Borisov, M. Romeo, F. Scheurer, J. Aizpurua, and G. Schull, Mapping Lamb, Stark, and Purcell effects at a chromophore-picocavity junction with hyper-resolved fluorescence microscopy, Phys. Rev. X **12**, 011012 (2022).

[4] M. J. Frisch, G. W. Trucks, H. B. Schlegel, G. E. Scuseria, M. A. Robb, J. R. Cheeseman, G. Scalmani, V. Barone, G. A. Petersson, H. Nakatsuji, X. Li, M. Caricato, A. V. Marenich, J. Bloino, B. G. Janesko, R. Gomperts, B. Mennucci, H. P. Hratchian, J. V. Ortiz, A. F. Izmaylov, J. L. Sonnenberg, D. Williams-Young, F. Ding, F. Lipparini, F. Egidi, J. Goings, B. Peng, A. Petrone, T. Henderson, D. Ranasinghe, V. G. Zakrzewski, J. Gao, N. Rega, G. Zheng, W. Liang, M. Hada, M. Ehara, K. Toyota, R. Fukuda, J. Hasegawa, M. Ishida, T. Nakajima, Y. Honda, O. Kitao, H. Nakai, T. Vreven, K. Throssell, J. A. Montgomery, Jr., J. E. Peralta, F. Ogliaro, M. J. Bearpark, J. J. Heyd, E. N. Brothers, K. N. Kudin, V. N. Staroverov, T. A. Keith, R. Kobayashi, J. Normand, K. Raghavachari, A. P. Rendell, J. C. Burant, S. S. Iyengar, J. Tomasi, M. Cossi, J. M. Millam, M. Klene, C. Adamo, R. Cammi, J. W. Ochterski, R. L. Martin, K. Morokuma, O. Farkas, J. B. Foresman, and D. J. Fox, Gaussian 16 revision C.01 (2016).

[5] A. D. Becke, Density-functional thermochemistry. iii. the role of exact exchange, J. Chem. Phys. **98**, 5648 (1993).

[6] R. Temirov, M. F. B. Green, N. Friedrich, P. Leinen, T. Esat, P. Chmielniak, S. Sarwar, J. Rawson, P. Kögerler, C. Wagner, M. Rohlfing, and F. S. Tautz, Molecular model of a quantum dot beyond the constant interaction approximation, Phys. Rev. Lett. **120**, 206801 (2018).

[7] M. Žonda, O. Stetsovych, R. Korytár, M. Ternes, R. Temirov, A. Raccanelli, F. S. Tautz, P. Jelínek, T. Novotný, and M. Švec, Resolving ambiguity of the Kondo temperature determination in mechanically tunable single-molecule Kondo systems, J. Phys. Chem. Lett. **12**, 6320 (2021).

[8] COMSOL multiphysics v. 5.5., COMSOL AB, Stockholm, Sweden (2022).

[9] E. V. Chulkov, V. M. Silkin, and P. M. Echenique, Image potential states on metal surfaces: binding energies and wave functions, Surf. Sci. **437**, 330 (1999).

[10] R. Kosloff, The fourier method, in *Numerical Grid Methods and Their Application to Schrödinger's Equation* (Springer, 1993) pp. 175–194.
[11] C. Leforestier, R. Bisseling, C. Cerjan, M. Feit, R. Friesner, A. Guldberg, A. Hammerich, G. Jolicard, W. Karrlein, H.-D. Meyer, N. Lipkin, O. Roncero, and R. Kosloff, A comparison of different propagation schemes for the time dependent schrödinger equation, J. Comput. Phys. **94**, 59 (1991).
[12] X. Gonze, J.-M. Beuken, R. Caracas, F. Detraux, M. Fuchs, G.-M. Rignanese, L. Sindic, M. Verstraete, G. Zerah, F. Jollet, M. Torrent, A. Roy, M. Mikami, P. Ghosez, J.-Y. Raty, and D. Allan, First-principles computation of material properties: the ABINIT software project, Comput. Mater. Sci. **25**, 478 (2002).
[13] J. P. Perdew and Y. Wang, Accurate and simple analytic representation of the electron-gas correlation energy, Phys. Rev. B **45**, 13244 (1992).
[14] O. T. Hofmann, V. Atalla, N. Moll, P. Rinke, and M. Scheffler, Interface dipoles of organic molecules on Ag(111) in hybrid density-functional theory, New J. Phys. **15**, 123028 (2013).
[15] H. Sun, S. Ryno, C. Zhong, M. K. Ravva, Z. Sun, T. Körzdörfer, and J.-L. Brédas, Ionization energies, electron affinities, and polarization , J. Chem. Theory Comput. **12**, 2906 (2016).
[16] T. Koopmans, Über die Zuordnung von Wellenfunktionen und Eigenwerten, Physica **1**, 104 (1934).
[17] L. Kleinman and D. M. Bylander, Efficacious form for model pseudopotentials, Phys. Rev. Lett. **48**, 1425 (1982).
[18] P. J. Jennings, R. O. Jones, and M. Weinert, Surface barrier for electrons in metals, Phys. Rev. B **37**, 6113 (1988).
[19] F. Aguilar-Galindo, A. G. Borisov, and S. Díaz-Tendero, Unveiling the anisotropic behavior of ultrafast electron transfer , Appl. Surf. Sci. **554**, 149311 (2021).
[20] U. V. Riss and H.-D. Meyer, Calculation of resonance energies and widths using the complex absorbing potential method, J. Phys. B: At. Mol. Opt. Phys. **26**, 4503 (1993).
[21] N. Moiseyev, Derivations of universal exact complex absorption potentials by the , J. Phys. B: At. Mol. Opt. Phys. **31**, 1431 (1998).
[22] A. Paulheim, C. Marquardt, M. Sokolowski, M. Hochheim, T. Bredow, H. Aldahhak, E. Rauls, and W. G. Schmidt, Surface induced vibrational modes in the fluorescence spectra of PTCDA adsorbed on the KCl(100) and NaCl(100) surfaces, PCCP **18**, 32891 (2016).
[23] K. Kimura, K. Miwa, H. Imada, M. Imai-Imada, S. Kawahara, J. Takeya, M. Kawai, M. Galperin, and Y. Kim, Selective triplet exciton formation in a single molecule, Nature **570**, 210 (2019).
[24] J. Doležal, S. Canola, P. Hapala, R. C. de Campos Ferreira, P. Merino, and M. Švec, Real space visualization of entangled excitonic states in charged molecular assemblies, ACS Nano **16**, 1082 (2022).
