# Supplementary material for: Fluorescence from a single-molecule probe directly attached to a plasmonic STM tip
Source: Nat Commun. 2024 Nov 10;15:9733. doi: 10.1038/s41467-024-53707-2 (PMC11551166; doi:10.1038/s41467-024-53707-2)
Supplement: Supplementary file 2 — Description of Additional Supplementary Files [file 41467_2024_53707_MOESM2_ESM.pdf]

Description of Additional Supplementary Files

File Name: Supplementary Data 1

Description: Experimental raw data.
