# Supplementary figures and images for: Fluorescence from a single-molecule probe directly attached to a plasmonic STM tip
Source: Nat Commun. 2024 Nov 10;15:9733. doi: 10.1038/s41467-024-53707-2 (PMC11551166; doi:10.1038/s41467-024-53707-2)

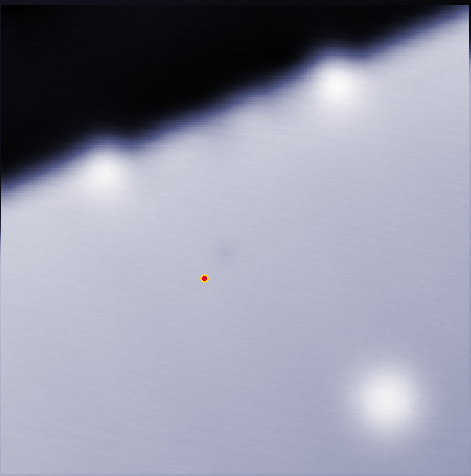

Supplement: Supplementary file 3 — Supplementary Data 1 [file 41467_2024_53707_MOESM3_ESM.zip › Fig3/Linescan1_Pos1.PNG]

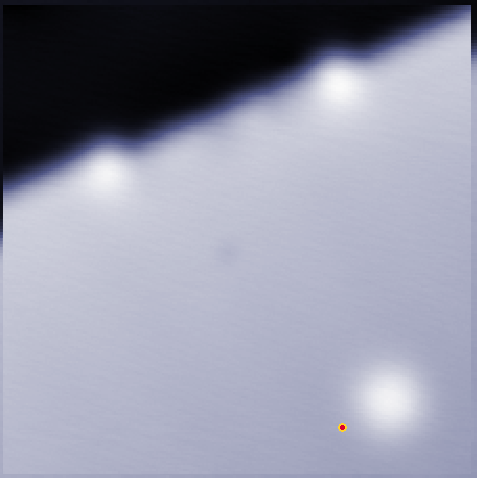

Supplement: Supplementary file 3 — Supplementary Data 1 [file 41467_2024_53707_MOESM3_ESM.zip › Fig3/Linescan1_Pos10.PNG]

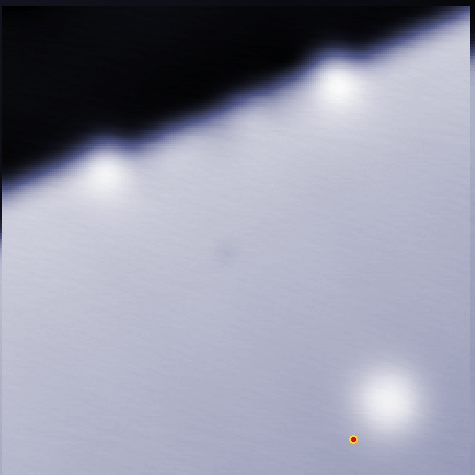

Supplement: Supplementary file 3 — Supplementary Data 1 [file 41467_2024_53707_MOESM3_ESM.zip › Fig3/Linescan1_Pos11.PNG]

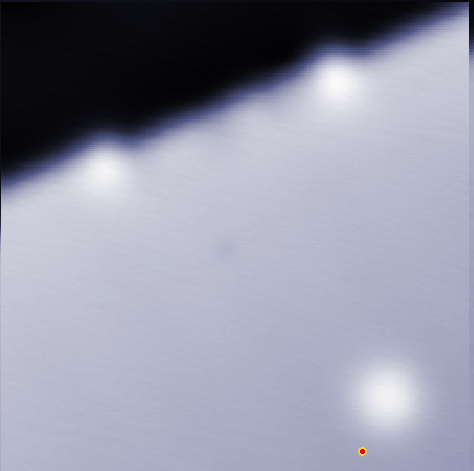

Supplement: Supplementary file 3 — Supplementary Data 1 [file 41467_2024_53707_MOESM3_ESM.zip › Fig3/Linescan1_Pos12.PNG]

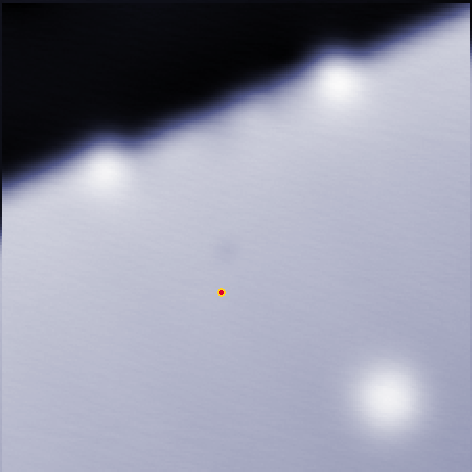

Supplement: Supplementary file 3 — Supplementary Data 1 [file 41467_2024_53707_MOESM3_ESM.zip › Fig3/Linescan1_Pos2.PNG]

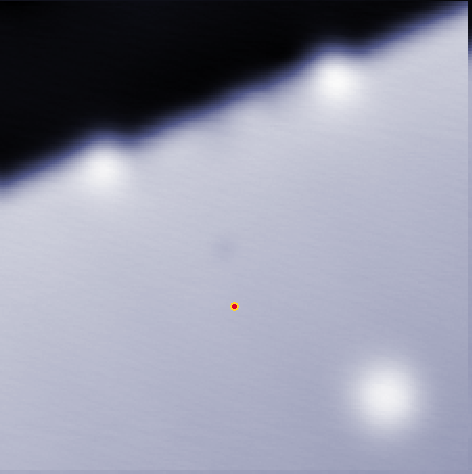

Supplement: Supplementary file 3 — Supplementary Data 1 [file 41467_2024_53707_MOESM3_ESM.zip › Fig3/Linescan1_Pos3.PNG]

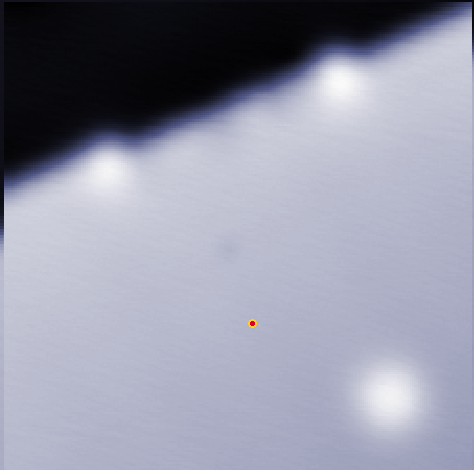

Supplement: Supplementary file 3 — Supplementary Data 1 [file 41467_2024_53707_MOESM3_ESM.zip › Fig3/Linescan1_Pos4.PNG]

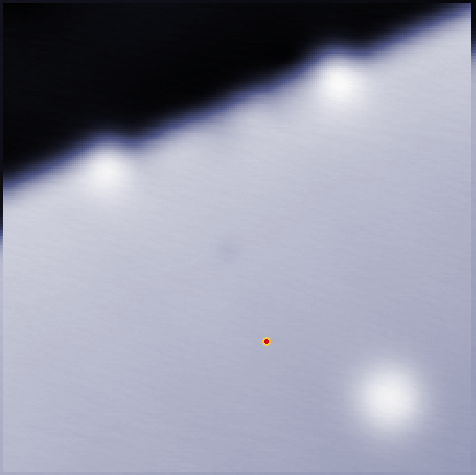

Supplement: Supplementary file 3 — Supplementary Data 1 [file 41467_2024_53707_MOESM3_ESM.zip › Fig3/Linescan1_Pos5.PNG]

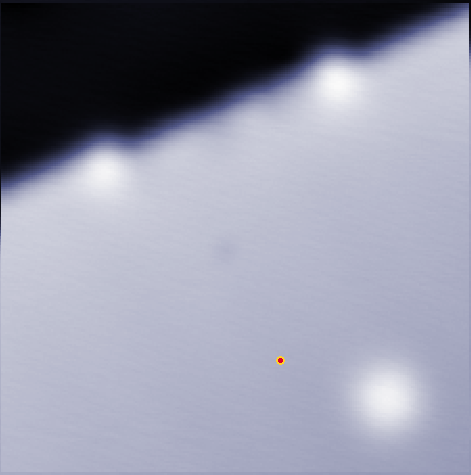

Supplement: Supplementary file 3 — Supplementary Data 1 [file 41467_2024_53707_MOESM3_ESM.zip › Fig3/Linescan1_Pos6.PNG]

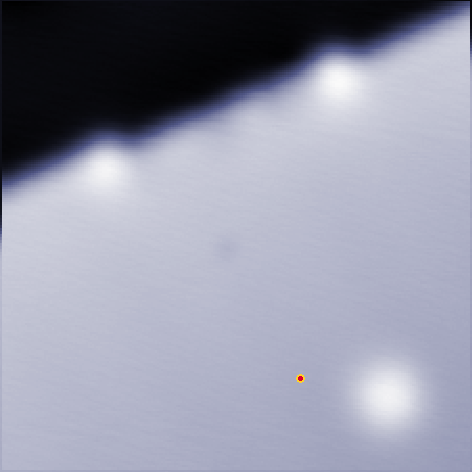

Supplement: Supplementary file 3 — Supplementary Data 1 [file 41467_2024_53707_MOESM3_ESM.zip › Fig3/Linescan1_Pos7.PNG]

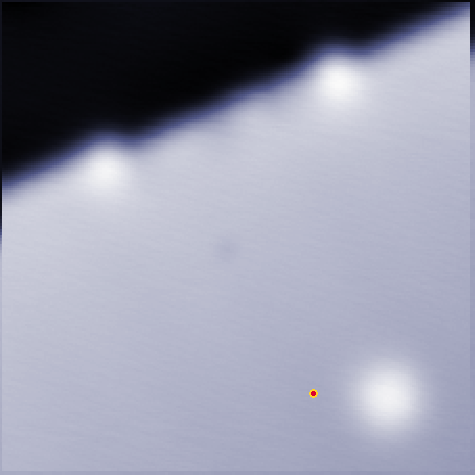

Supplement: Supplementary file 3 — Supplementary Data 1 [file 41467_2024_53707_MOESM3_ESM.zip › Fig3/Linescan1_Pos8.PNG]

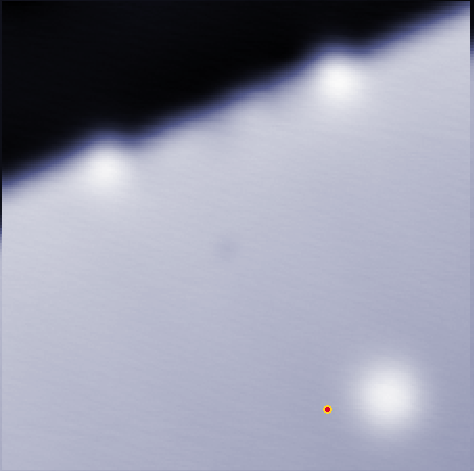

Supplement: Supplementary file 3 — Supplementary Data 1 [file 41467_2024_53707_MOESM3_ESM.zip › Fig3/Linescan1_Pos9.PNG]
